# Sleep movements and respiratory coupling as a biobehavioral metric for early Alzheimer’s disease in independently dwelling adults

**DOI:** 10.1186/s12877-023-03983-2

**Published:** 2023-04-27

**Authors:** Somayeh Khosroazad, Christopher F. Gilbert, Jessica B. Aronis, Katrina M. Daigle, Masoumeh Esfahani, Ahmed Almaghasilah, Fayeza S. Ahmed, Merrill F. Elias, Thomas M. Meuser, Leonard W. Kaye, Clifford M. Singer, Ali Abedi, Marie J. Hayes

**Affiliations:** 1 Electrical and Computer Engineering, University of Maine, 5708 Barrows Hall, Orono, ME 04469 USA; 2 Activas Diagnostics, LLC, 20 Godfrey Dr., Orono, ME 04473 USA; 3 Psychology Department, University of Maine, 5740 Beryl Warner Williams Hall, Orono, ME 5740-04469 USA; 4 Psychology Department, Suffolk University, 73 Tremont St., Boston, MA 02108 USA; 5 Graduate School of Biomedical Science & Engineering, University of Maine, 5775 Stodder Hall, Orono, ME 04469 USA; 6 Center for Excellence On Aging, University of New England, 11 Hills Beach Rd., Biddeford, ME 04005 USA; 7 Center On Aging, University of Maine, 327 Camden Hall, Orono, ME 04469 USA; 8 Mood and Memory Clinic, Northern Light Health, 269 Stillwater Ave., Bangor, ME 04402 USA

**Keywords:** Alzheimer’ Disease and Related Disorders (ADRD), Mild Cognitive Impairment, Amnestic type (aMCI), Sleep disorder, Memory loss, Aging, Home sleep device, Sleep movements, Arousal system, Respiration, Artificial intelligence

## Abstract

**Introduction:**

Sleep disorder is often the first symptom of age-related cognitive decline associated with Alzheimer’s disease (AD) observed in primary care. The relationship between sleep and early AD was examined using a patented sleep mattress designed to record respiration and high frequency movement arousals. A machine learning algorithm was developed to classify sleep features associated with early AD.

**Method:**

Community-dwelling older adults (*N* = 95; 62–90 years) were recruited in a 3-h catchment area. Study participants were tested on the mattress device in the home bed for 2 days, wore a wrist actigraph for 7 days, and provided sleep diary and sleep disorder self-reports during the 1-week study period. Neurocognitive testing was completed in the home within 30-days of the sleep study. Participant performance on executive and memory tasks, health history and demographics were reviewed by a geriatric clinical team yielding Normal Cognition (*n* = 45) and amnestic MCI-Consensus (*n* = 33) groups. A diagnosed MCI group (*n* = 17) was recruited from a hospital memory clinic following diagnostic series of neuroimaging biomarker assessment and cognitive criteria for AD.

**Results:**

In cohort analyses, sleep fragmentation and wake after sleep onset duration predicted poorer executive function, particularly memory performance. Group analyses showed increased sleep fragmentation and total sleep time in the diagnosed MCI group compared to the Normal Cognition group. Machine learning algorithm showed that the time latency between movement arousals and coupled respiratory upregulation could be used as a classifier of diagnosed MCI vs. Normal Cognition cases. ROC diagnostics identified MCI with 87% sensitivity; 89% specificity; and 88% positive predictive value.

**Discussion:**

AD sleep phenotype was detected with a novel sleep biometric, time latency, associated with the tight gap between sleep movements and respiratory coupling, which is proposed as a corollary of sleep quality/loss that affects the autonomic regulation of respiration during sleep. Diagnosed MCI was associated with sleep fragmentation and arousal intrusion.

Age increases the risk for mild cognitive impairment (MCI), or early dementia, which may impact memory and/or non-memory cognitive domains but typically spares functional abilities [1, 2]. MCI is linked to genetic vulnerability (APOE4), lifestyle and associated comorbidities (e.g., cardiovascular disease, traumatic brain injury, socioeconomic status (SES), education, obesity, depressive symptoms, etc.) [2, 3]. Early Alzheimer’s disease accounts for approximately 50% of MCI incidence [4] and is characterized by cognitive decline in memory domains (amnestic type); whereas, vascular dementia, the second most common subtype of dementia with an incidence of 14.5%, is characterized by deficits in non-memory cognitive domains (e.g. visuospatial skills, attention, language, etc.) without memory impairment (nonamnestic type, [5, 6]). A nongradual progression of cognitive deficits is consistent with neurodegenerative disease [7].

## Biomarkers in early Alzheimer’s disease

In 2018, the National Institute on Aging and Alzheimer’s Association Research Framework, updated the definition and diagnostic process to identify Alzheimer’s disease (AD) using pathology markers documented in vivo or at postmortem examination [8]. The advancement in biomarker diagnostic decision making moves away from a syndromal approach (i.e., symptoms and behavioral deficits) to a biological approach for staging AD and other neurological diseases. The understanding of beta amyloid (Aβ) and tau peptides as biomarkers in the CSF (Aβ42, Aβ40, t-tau, p-tau) in AD development has led to the AT(N) scoring system for neuroimaging assessment of AD and other neurological disease: A indicates PET ligand binding of Aβ plaques or low CSFAb42, T, CSF phosphorylated tau, N, neurodegeneration or injury (N) [9, 10]. Current progress in detection of AD and other neurological disease depends on the presence of Aβ with or without tau or neurodegeneration in CNS and plasma biomarkers of disease [11, 12], although p-tau plasma biomarker, NF1 [13] and p-tau217 [14] show promise in longitudinal studies for early AD. The preclinical phase based on biomarker positivity in CSF and PET, occurs prior to the onset of symptoms and is longer than the time of age proposed in MCI-AD. A previous preclinical period is not always observed in vascular MCI etiology [15].

## Sleep, memory and AD

Sleep is an example of another area of functional change in aging that is associated with cognitive loss [16]. Recent animal and human studies of Aβ metabolism support the relationship between sleep quality, cycles of Aβ clearance and AD pathogenesis [17, 18]. The long pre-dementia phase for aMCI is approximately 6–10 years, and results from prospective studies have identified sleep disorder as a frequent symptom linked with disease progression [15]. A critical question is whether sleep abnormalities are present early in the process.

In community dwelling aging adults, disturbances in sleep and circadian rhythms correlate with early signs of neurodegeneration. Several studies in community aging samples have shown that poor sleep quality and chronic sleep loss are associated with cognitive decline [19–21]. Recent community studies found that poorer sleep quality and shorter or longer sleep duration was associated with greater Aβ burden in the cortical and precuneus areas, and greater cortical thinning [22, 23], supporting an association consistent with preclinical AD and sleep disorder. AD risk is increased in persons > 60 years of age if sleep duration dips below 6 h/day which leads to chronic sleep loss [24]. Untreated sleep disorder rapidly converts to chronic sleep deprivation linked to increased soluble Aβ [25], as well as other AD and biomarkers in the CSF (Aβ42, Aβ40, t-tau, p-tau, and chitinase-3-like protein1) which is reversed by sleep extension in animal models of aging [26, 27]. In AD-related sleep disorder, the hypothesis is that sleep deprivation potentially drives disease progression, and that sleep loss is cumulative and associates with circadian dysregulation and cognitive impairment in the conversion to the AD phenotype [27, 28].

Memory-related neuroplasticity requires stable sleep continuity to achieve memory consolidation. NREM and REM sleep stages are critical for memory consolidation, a selective domain deficit in aMCI and AD [29, 30]. MCI sleep complaints are detected in some actigraphy studies in the form of sleep disturbances such as wake after sleep onset (WASO); arousals/hour of sleep; and poor sleep stage stability [21, 23]. EEG deficits in fast-frequency sleep spindle deficits are magnified in patients with MCI and AD [31, 32]. In normal aging and middle-aged adults, sleep spindles decrease non-linearly with age and spindle cluster size predict memory consolidation, an effect that was disrupted by sleep fragmentation. Benca and colleagues have found that in AD-enriched, cognitively unimpaired patients spindle loss correlated with overnight memory deficits, and was associated with increased microglia activation, phosphorylated tau and synaptic degeneration [33].

Chronic sleep loss over time suppresses arousability during sleep epochs, reduces daytime alertness, compromising cognitive functioning in a dose dependent manner, and impairs circadian timing systems [34]. Importantly, autonomic changes also occur in cardiorespiratory regulation during sleep that emerge with chronic sleep loss [35, 36]. In our prior work, which forms the basis of the current study, we have observed that reduced arousability from chronic sleep deprivation can be monitored by recording micro-sleep movements (SM) which are periodic (circa 2 min) and decrease in amplitude and duration during sleep loss such as when CNS injury or disease is present [37–40]. The CNS arousal system is dampened during chronic sleep loss, which similarly affects periodic micro-sleep movements, supporting this measure as or part of the arousal system. The patented mattress sleep device is uniquely sensitive to rapid, low impact movement events not detected by actigraphy, and often ruled-out in polysomnography as noise. We report in the current study that the periodic (circa 2 min) property of the sleep movement arousal system (SM) is coupled to respiratory frequency upregulation during normal sleep.

The first hypothesis of the present study examines the sleep-cognitive link in our aging community cohort. We hypothesized that impaired sleep quality (assessed using actigraphy) is associated with reduced cognitive performance in our community cohort. Cognitive performance tested across domains was compared to sleep measures across all participants.

The second hypothesis proposes that the novel biometric from the sleep device system (i.e. time latency between coupled movement and respiratory events) could be used as a possible risk signature of early AD. The clinical goal is to ask whether this biometric, acquired through noninvasive home-bed recordings, may assist in the evaluation of aging patients’ complaints of sleep loss and/or cognitive problems to prepare referral for AD and other biomarker testing for differential diagnosis.

## Method

### Participants

**Fig. 1:**
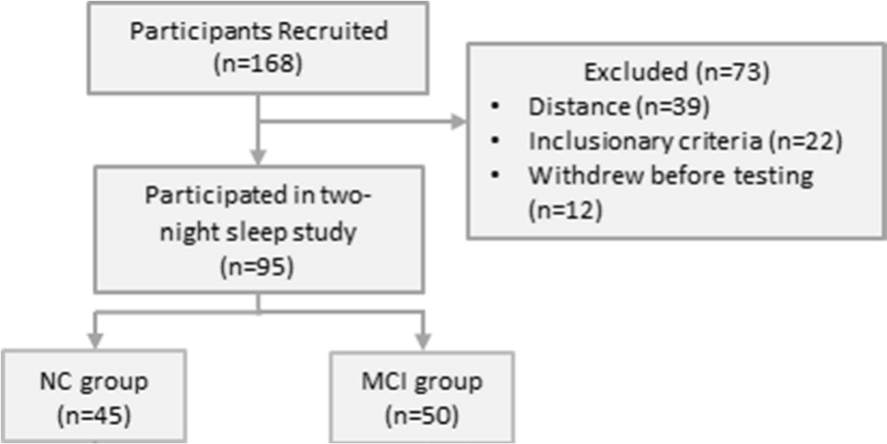
Recruitment flowchart shows that of 168 recruited participants, 73 were excluded based on exclusionary criteria; 39 because of distance from the laboratory; 12 participants withdrew from the study. Diagnosed MCI patients (*n* = 17) were referred by C.S. from the Mood and Memory Center, Acadia Hospital, Bangor, ME and are included

The *Maine Sleep and Aging Study* was conducted by the University Maine. The experimental protocol was approved by University of Maine Institutional Review Board for Research Ethics (Federalwide Assurance #: FWA00000479; IRB Organization (IORG) #: IORG0000642). All experimental protocols were approved by this IRB and all methods were carried out in accordance with relevant guidelines and regulations of this ethics committee. Independent community dwelling, aging adults were sought through advertisement within a 3-h catchment area. Inclusionary Criteria: Participants were between 60–90 years of age, lived independently in the community, were English speaking and possessed adequate vision with correction. Exclusionary Criteria: Any medical evidence through exam or imaging of a neurological, psychiatric or medical disorder other than AD as a cause for MCI (e.g. partial list: more than one cerebral infarct, poorly controlled diabetes, hypothyroidism, parkinsonism, parasomnia or REM sleep disorder, developmental disability, schizophrenia, etc.); acute symptom onset; depressive symptoms score on CES-D ≥ 16 (Center for Epidemiological Studies Depression Scale); Epworth Sleepiness Scale score ≥ 10; restless or periodic leg syndrome; hypnotic/ psychotropic medication change [37]. History of depression/anxiety disorders or > 5 years drug/ alcohol abuse but in recovery was allowed. Figure 1 illustrates that 168 participants were recruited and 73 were excluded based on distance (*n* = 22), exclusionary criteria (*n* = 39), or withdrawal or failure to schedule (*n* = 12). The final sample of 95 participants completed the home sleep testing protocol.

Included in the sample was a clinic-diagnosed aMCI patient group (MCI-DX; *n* = 17) recruited from Northern Light Healthcare, Bangor, ME by co-author, C.S., according to the criteria above and including a complete medical review. The diagnosed MCI (MCI-DX) group included subjective and family complaints of memory problems, and a review of other disease sources of symptomatology. Bio- banked plasma was collected for eventual diagnostic confirmation of plasma p-tau/Aβ42 when funds become available.

The remaining cohort was evaluated for aMCI diagnosis by an expert consensus panel (co-authors C.S., F.A., J.A.) using demographics, full battery of neurocognitive testing, and comorbidities (MCI-Consensus group, MCI-CON; *n* = 33). MCI-CON group met aMCI criteria for immediate and delayed verbal and visual memory tasks, but were normative on intelligence and cognitive reserve measures using Petersen [26] and the DSM-5 [4] criteria; Montreal Cognitive Assessment (MoCA) score of 26–19 (inclusive); delayed recall subtest score of 0 or 1 out of 5; normal circadian entrainment to nighttime sleep hours; and decision-making capacity to independently consent to research. The consensus panel distinguished aMCI (MCI-CON; *n* = 33) and normal cognition groups (NC; *n* = 45). Vascular or mixed MCI were found in two cases and were exclusionary.

## Instrumentation and measures

### SleepMove mattress device and system

The wireless sensor mattress pad is 1 mm in thickness within a sealed polyethylene cover that is placed within an antimicrobial-resistant fitted sheet positioned beneath the bottom bed sheet on the home bed for 2 nights. The sensor device design uses 32 distributed piezo-resistive pressure sensors to record movement and respiratory signals using 54 Hz average sampling rate. The receiver unit under the bed records data to a Micro SD card. System algorithms filter sleep movement (SM) bouts (periodicity circa 0.198 Hz) and respiration (periodicity circa 0.366 Hz) into two data streams. Time series algorithms identify SM-RR coupling using a concordance estimate of time latency (ms) to capture the time delay between SM and RR, thereby calculating the strength of the coupling relationship between these two events. Respiratory frequency is indexed by segmentation of RR rate into 2 min bins. These data form the basis of artificial intelligence (AI) analyses described herein and in technical detail in Khosroazad, Abedi & Hayes (in press) [41].

In a standard laboratory and home settings, we validated concordance of the respiratory inspiratory and expiratory cycle and periodicity of standard thermistor recordings during sleep contrasted with mattress device data. Mattress-derived movement arousals, SM features and periodicity have been confirmed through videosomnography in prior work [37–43].

### Actigraphy

The participants wear Actiwatch 2 (Philips Respironics, Philips Actiware 6: v.6.0.9), a watch-like actigraphy monitor on the non-dominant wrist for seven consecutive nights beginning on night 1 of the study. Actigraphy software provides standard sleep–wake measures.

### Self-report

Stanford Sleepiness Scale: queries subjective sleepiness with a 7-point Likert scale for 7 consecutive days [44]; Epworth Sleepiness Scale: asks about daytime sleepiness and situations [45]; Pittsburgh Sleep Quality Index (PSQI): assesses sleep quality over a 1-month time interval with several subscales and a composite score [46]. Center for Epidemiological Studies Depression Scale (CES-D): 10-item depression scale used for older adults [47].

### Neurocognitive testing

Montreal Cognitive Assessment (MoCA) screening tool for MCI that includes short term and working memory, visuospatial, attention, orientation and executive functioning (range score for MCI is 19–25) [48]. Brief Visuospatial Memory Test-Revised (BVMT-R): [49]. Hopkins Verbal Learning Test-Revised (HVLT-R) [50]. Boston Naming Test (BNT): confrontation naming task [51]. Proxies for cognitive reserve were measured by the Vocabulary subtest of the Wechsler Adult Intelligence Scale (WAIS III; [52]), and American National Adult Reading test (AMNART; [53]), both of which are well-established means of estimating cognitive reserve.

### Protocol

**Fig. 2:**
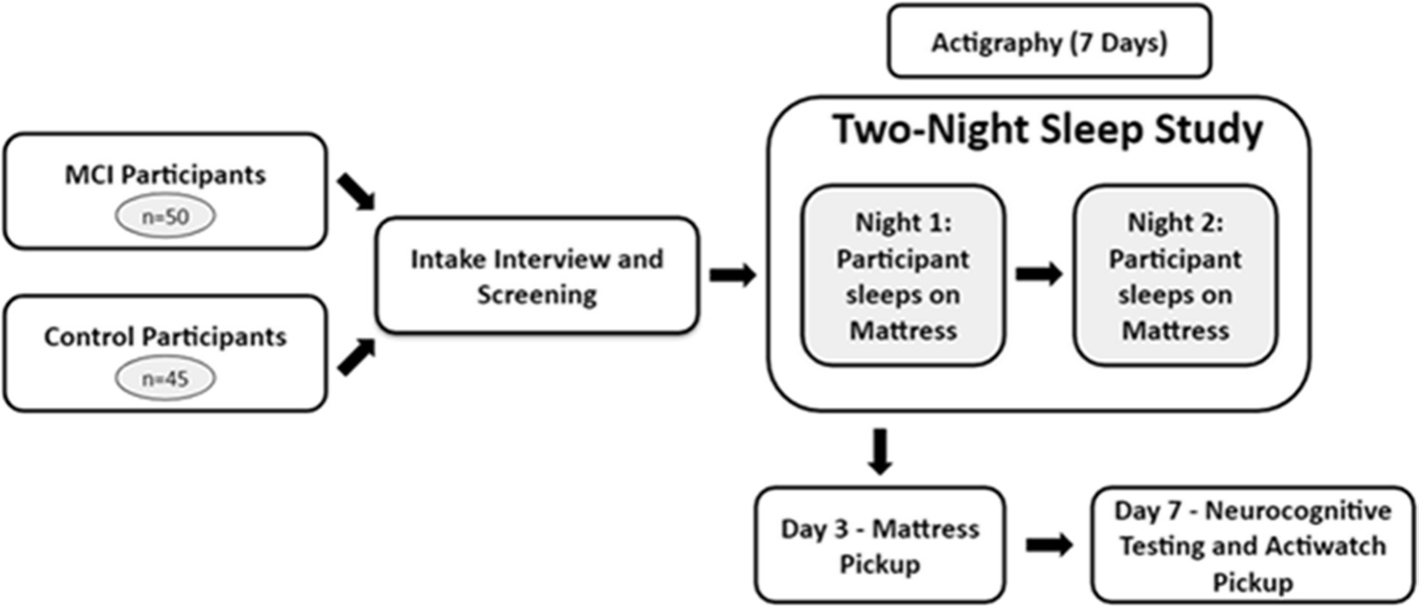
Protocol for the home sleep study with participants. SleepMove mattress device was delivered and positioned by the research staff, and participants were interviewed following informed consent. Seven days of actigraphy overlapped with the 2 day SleepMove study, and participants completed questionnaires on sleep problems

Figure 2 shows the testing sequence. Participant eligibility was determined by phone interview screening. On study day 1 (night 1), two team members arrived at the home at approximately 1700 h to interview the participant; administer the MoCA and query: 1.) medical comorbidities; 2.) SES (socioeconomic status) through longest held career, e.g., secretarial, professional, homemaker, etc.; 3.) lifestyle and substance use using current and former intake of alcohol, tobacco and other use; weekly exercise and activities; 4.) obesity risk estimated through Body Mass Index; and 4.) self-report inventories of sleep problems (see Instrumentation and Measures).

The participants slept alone in the home bed on the mattress device for 2 consecutive nights. The signal acquisition hardware was placed under the bed connected to the mattress pad using a flat wire powered by battery. After night 2, the device was picked up and data transferred electronically to the laboratory. Participants reported bed and wake times and wore the actiwatch for 7 days. Neurocognitive testing was conducted in a home visit for approximately 90 min. The participant received a $100 Visa gift card for participation.

### Statistics and data processing

Multivariable, mixed model regression using group as a factor were used for data analysis for actigraphy, neurocognitive and sleep self-report data moderated by custom covariates as described (e.g., age, education, BMI) with IBM-SPSS, V.26. Demographics were analyzed with one-way ANOVA for continuous variables and Kruskal–Wallis h test for categorical variables. Imputation was used in < 5% of the data.

For the SM-RR coupling output measure, a supervised Neural Network approach was used. SM and RR signals were separated in the frequency domain using FFT and band pass frequency filters as described in Instrumentation and Measures. SM-RR coupling time latency was examined in 10 min windows to determine the Probability Density Function (PDF) of the maximum time latencies for each case. Receiver Operating Characteristic (ROC) statistics were applied to determine sensitivity and specificity, AUC (area under the curve) and c statistic with confidence interval. ROC plot illustrates the diagnostic binary classifier system, in this case, MCI vs. NC as its discrimination threshold is varied. Positive predictive values and negative predictive values were calculated to identify MCI status using sensitivity (true positives) and specificity (false positives). Neural Network and the cross-validation method, Leave-One-Out Covariance (LOOCV), was used to train and test the network [54]. Confusion matrices of true positive, true negative, false positive and false negative were calculated based on the verification group that was placed in an incorrect category by the trained network.

## Results

### Demographics

Table 1 displays cross-group findings for health and demographic data. ANOVA revealed small but significant differences in age (F(2, 94) = 3.39, *p* < 0.038, η^2^ = 0.069). In post hoc testing, MCI-DX group was significantly older than the NC group (*p* < 0.013). MoCA scores also differed by group in ANOVA analyses (F(2, 94) = 56.51, *p* < 0.001, η^2^ = 0.551). Both MCI-DX and MCI-CON groups were in the diagnostic range (score range 19–24), although MCI-DX scores were significantly lower than MCI-CON (*p* < 0.001), and both MCI groups were significantly lower than MCI-NC (*p* < 0.001). Body Mass Index (BMI), a known risk for MCI/AD [39], differed by group (F(2, 89) = 3.13, *p* < 0.049, η^2^ = 0.067), and was significantly higher in MCI-CON than MCI-DX (*p* < 0.015). In nonparametric analyses (Kruskal–Wallis H = 4.41, *p* < 0.036, η^2^ = 0.027), both MCI groups (MCI-DX = 46.7%, MCI-CON = 33.3%) were significantly more likely to report current depressive symptoms than the NC group (13%; *p* < 0.038).

**Table 1:** Demographics and Health History in MCI-DX, MCI-CON, and NC Groups

Variable (µ ± SE or % (n))	MCI-DX (*n* = 17)	MCI-CON (*n* = 33)	NC (*n* = 45)	*p*-value
Age, y	75.94 ± 1.39	73.42 ± 1.24	71.31 ± 0.91	**0.038**
Female	70.6% (12)	60.6% (20)	77.8% (35)	n.s
Race (% white)	100% (17)	100% (33)	97.8% (44)	n.s
Years of education	15.07 ± 0.84	15.55 ± 0.53	15.60 ± 0.35	n.s
MoCA	19.76 ± 0.78	22.45 ± 0.48	26.73 ± 0.30	**0.001**
**Lifestyle factors**				
Drinking alcohol	33.3% (5)	66.7% (22)	62.2% (28)	n.s
Current use of sleep medication^**a**^	60% (9)	33.3% (11)	42.2% (19)	n.s
Current or former smoker	53.3% (8)	54.5% (18)	46.7% (21)	n.s
**Body mass index (BMI)**	24.65 ± 1.38	29.37 ± 1.09	27.60 ± 0.86	**0.049**
**OSA**	11.8% (2)	36.4% (12)	17.8% (8)	n.s
**Diabetes**	6.7% (1)	27.3% (9)	11.1% (5)	n.s
**Heart attack or cardiac arrest**	20% (3)	12.1% (4)	11.1% (5)	n.s
**Cardiovascular disease**	6.7% (1)	24.2% (8)	13.3% (6)	n.s
**Cerebrovascular disease**	33.3% (5)	9.1% (3)	6.7% (3)	n.s
**Traumatic brain injury (TBI)**	26.7% (4)	15.2% (5)	15.6% (7)	n.s
**Hypercholesterolemia**	53.3% (8)	36.4% (12)	42.2% (19)	n.s
**Hypertension**	33.3% (5)	54.5% (18)	34.1% (15)	n.s
**Arthritis**	46.7% (7)	69.7% (23)	48.9% (22)	n.s
**Thyroid disease**	40% (6)	18.2% (6)	33.3% (15)	n.s
**Current depressive symptoms**^**b**^	46.7% (7)	33.3% (11)	13.3% (6)	**0.036**

*Note:* Values are shown as % (n) or mean ± SE, and compared by one-way ANOVA (continuous variables) or Kruskal–Wallis Test (categorical variables). *MCI-DX* Physician diagnosed, *MCI-CON* Expert panel consensus decision based on clinical status and neuro-cognitive assessments, *NC* Normal cognition groups. ^a^Current use of sleep medication was described as any self-reported use of over the counter or prescribed sleep medication over the last month. ^b^ Current depressive symptoms were indicated by self-report answer to the question “Are you currently feeling depressed?”

### Neurocognitive performance

aMCI diagnosis criteria require threshold-defined deficits in immediate and delayed verbal and visuospatial memory domains with normative scores on cognitive reserve and intelligence measures. The clinical criterion of cognitive performance > 1.5 S.D. below age-adjusted norms as the standard for cognitive impairment revealed that 16% of the full cohort had scores at this level, and all were from MCI-DX group. MCI-DX group (*n* = 17) repeated the neurocognitive protocol for this study although formal diagnostic evaluation had been conducted in the clinic. MCI-CON and NC groups’ decisions were based on the consensus panel review.

In Table 2, multivariable regression was used to examine cognitive performance with group as a factor and age and current depressive symptoms as covariates. The results confirmed the expected severity stacking of MCI-DX, MCI-CON and NC groups showing that MCI groups have selective memory domain deficits reflected in Hopkins Verbal Learning Test, revised (HVLT-R) and Brief Visuospatial Memory Test, revised (BVMT-R) measures consistent with a selective amnestic cognitive burden in these groups. Regression findings for HVLTR-R-total recall (F (10, 93) = 246.6, *p* < 0.001, η2 = 0.967) confirmed the group main effect. Post-hoc testing found that MCI-DX (*p* < 0.001) and MCI-CON (*p* < 0.001) performed significantly worse than NC group, and MCI-DX performed significantly worse than MCI-CON (*p* < 0.002). HVLTR-delayed (F (10, 93) = 108.7, *p* < 0.001, η2 = 0.929) yielded the same group findings. MCI-DX (*p* < 0.001) and MCI-CON (*p* < 0.001) performed significantly worse than NC, and MCI-DX performed significantly worse than MCI-CON (*p* < 0.001). HVLTR-retention (F (10, 93) = 82.7, *p* < 0.001, η2 = 0.909) confirmed the group stacking: MCI-DX (*p* < 0.01) and MCI-CON (*p* < 0.003) performed worse than NC group, and MCI-DX retention scores were lower than MCI-CON (*p* < 0.016).

**Table 2:** MCI Severity and Neurocognitive Memory Domain Test Scores

**Neurocognitive Test**	**MCI Status**			**Ranking**
	**MCI-DX** **(***n* **= 17)**	**MCI-CON** **(***n* **= 33)**	**NC** **(***n* **= 45)**	**Post-hoc (p)**
HVLT-R Total Recall	13.57 (1.2)	17.95 (0.7)	23.68 (0.7)	MCI-DX < MCI-CON < NC (< 0.001–0.002)
HVLT-R Delayed Recall	2.59 (0.6)	5.11 (0.4)	8.54 (0.3)	MCI-DX < MCI-CON < NC (< 0.001–0.001)
HVLT-R Retention %	47.17 (7.7)	69.13 (4.6)	88.57 (4.4)	MCI-DX < MCI-CON < NC (< 0.003–0.016)
BVMT-R Trial 1	1.27 (0.5)	2.39 (0.3)	3.23 (0.3)	MCI-CON < NC (0.046); MCI-DX < NC (0.001)
BVMT-R Trial 2	1.90 (0.6)	4.25 (0.4)	5.45 (0.4)	MCI-DX < MCI-CON < NC (< 0.001–0.022)
BVMT-R Trial 3	1.83 (0.7)	5.69 (0.4)	7.14 (0.4)	MCI-DX < MCI-CON < NC (< 0.001–0.016)
BVMT-R Total Recall	4.61 (1.5)	12.32 (0.9)	15.78 (0.9)	MCI-DX < MCI-CON < NC (< 0.001–0.008)
BVMT-R Learning	1.70 (0.7)	3.4 (0.4)	4.07 (0.4)	MCI-DX < NC (0.002); MCI-DX < MCI-CON (0.028)
BVMT-R Delayed Recall	1.86 (0.8)	4.87 (0.4)	6.68 (0.4)	MCI-DX < MCI-CON < NC (< 0.001–0.005)
BVMT-R Retention %	64.19 (10.1)	84.5 (6.0)	88.81 (5.9)	MCI-DX < NC (0.037)
BNT Total Score	13.22 (0.4)	14.16 (0.2)	14.25 (0.2)	MCI-DX < MCI-CON (0.028)

*Note*. *HVLT-R* Hopkins Verbal Learning Test-Revised, *BVMT-R* Brief Visuospatial Memory Test-Revised, *BNT* Boston Naming Test. All neurocognitive variables are expressed as raw scores expressed as group means and standard error. Multivariable regression applied with group factor and neurocognitive tests dependent variables adjusted for age and depression was significant for all tests; post-hoc group comparisons are shown in parentheses. MCI-DX = Physician diagnosed MCI; MCI-CON = Expert panel consensus decision based on clinical status and neuro-cognitive assessments; NC = Normal cognition

Regression analyses of BVMT-R, trial 1 (F (12, 93) = 25.5, *p* < 0.001, η2 = 0.791) confirmed the group main effect. Post-hoc testing found that MCI-DX (*p* < 0.001) and MCI-CON (*p* < 0.046) performed significantly worse than NC group. BVMT-R, trial 2 (F (12, 93) = 48.3, *p* < 0.001, η2 = 0.877) revealed that MCI-DX (*p* < 0.001) and MCI-CON (*p* < 0.022) performed significantly worse than NC, and MCI-DX performed significantly worse than MCI-CON (*p* < 0.002). BVMTR-trial 3 (F (12, 93) = 58.25, *p* < 0.001, η2 = 0.896) showed MCI-DX (*p* < 0.001) and MCI-CON (*p* < 0.016) performed significantly worse than NC group, and MCI-DX scores were lower than MCI-CON (*p* < 0.001). BVMT-R, total recall (F (12, 93) = 63.2, *p* < 0.001, η2 = 0.904) revealed that MCI-DX (*p* < 0.001) and MCI-CON (*p* < 0.008) performed significantly worse than NC, and MCI-DX performed significantly worse than MCI-CON (*p* < 0.001). BVMTR-Learning (F (12, 93) = 21.8, *p* < 0.001, η2 = 0.764) analyses found that MCI-DX (*p* < 0.001), but not MCI-CON (n.s.), performed significantly worse than NC, and MCI-DX performed significantly worse than MCI-CON (*p* < 0.028). BVMTR-delayed recall (F (12, 93) = 43.7, *p* < 0.001, η2 = 0.866) showed that MCI-DX (*p* < 0.001) and MCI-CON (*p* < 0.005) performed significantly worse than NC, and MCI-DX performed significantly worse than MCI-CON (*p* < 0.001). BVMTR-percent retained (F (12, 93) = 82.8, *p* < 0.001, η2 = 0.884) showed that MCI-DX (*p* < 0.037), but not MCI-CON (n.s.), performed significantly worse than NC, while MCI-CON and MCI-DX groups’ post-hoc contrast was not significant. Boston Naming total score regression model was significant for group as well (F (8, 93) = 1504, *p* < 0.001, η2 = 0.993). Post-hoc pairwise comparisons showed that scores were lower for MCI-DX vs. NC groups (*p* < 0.028). As predicted, no significant group differences were found in WAIS III vocabulary subtest, the Boston Naming Test to test lexical retrieval, or the American National Reading Test (AMNART), for cognitive reserve*,* between groups.

### Sleep actigraphy and neurocognitive performance

As a manipulation check and to test the first hypothesis of the relationship of cognition to sleep in aging patients a cohort analysis was applied*.* In the full cohort, actigraphy and performance data were subjected to a multivariable regression model adjusted for age, current depressive symptoms, BMI, and education years. Table 3 shows the results of a tertile analysis that strongly associated poorer memory-based cognitive scores (i.e., HVLT-R and BVMT-R) with *sleep fragmentation*, a term for movement and wake intrusion during sleep and *wake after sleep onset* or WASO. Sleep fragmentation associated with poorer HVLT-R, total recall score (F (7, 82) = 193.8, *p* < 0.001, η2 = 0.945) due to a significant post hoc contrast for Tertile 2 > 3 (*p* < 0.009). Sleep fragmentation was associated with reduced performance on HVLT-R, delayed recall (F (7, 82) = 70.4, *p* < 0.001, η2 = 0.862) with significant post hoc contrast for Tertile 2 > 3 (*p* < 0.007). Similarly, WASO was associated with poorer performance in BVMT-R, trial 1 (F (7, 82) = 184.3, *p* < 0.001, η2 = 0.942) with post hoc contrast showing Tertile 1 > 3 (*p* < 0.017). WASO was associated with impairment in HVLT-R raw total recall (F (7, 82) = 34.6, *p* < 0.001, η2 = 0.862, showing a trend for Tertile1 > 3 (*p* < 0.057). As predicted, cognitive reserve and intelligence assessments, AMNART, BNT and WAIS-III subtest, did not associate with these or other autography metrics.

**Table 3:** Neurocognitive Performance and Sleep Fragmentation (SF) and WASO

Neurocognitive Test		Unadjusted [p (95% CI)]	Adjusted [p (95% CI)]	Post-hoc
**SF**^a^	^a^HVLT-R Raw (total recall)	0.004 (1.37, 7.15)	0.009 (0.95, 6.36)	Tertile 2 > Tertile 3
	HVLT-R Raw (delayed recall)	0.004 (0.81, 4.02)	0.008 (0.54, 3.56)	Tertile 2 > Tertile 3
**WASO**^b^	BVMT-R Raw (trial 1)	0.013 (0.27, 2.24)	0.014 (0.25, 2.14)	Tertile 1 > Tertile 3
	HVLT-R Raw (total recall)	0.044 (0.09, 6.01)	0.057 (-0.09, 5.66)	Tertile 1 > Tertile 3

Multivariable regression of significant actigraphy predictors of neurocognitive performance adjusted for age, current depressive symptoms, BMI, and education years. SF/HVLT-R (total recall): F(7,82) = 193.8, *p* < .001, η2 = .945; SF/HVLT-R (delayed recall): F(7,82) = 70.4, *p* < .001, η2 = .862. WASO/BVMTR (trial 1): F(7, 82) = 184.3., *p* < .001, η2 = .942; WASO/HVLTR-R(total recall): F(7,82) = 34.6, *p* < .001, η2 = .862

^a^Tertile 1: *n* = 29; mean = 21.33 ± 3.51 counts, 12.83 to 25.89; Tertile 2: *n* = 29; mean = 32.59 ± 3.63 counts, 26.15 to 38.96; Tertile 3: *n* = 31; mean = 51.63 ± 11.72 counts, 39.40 to 93.13

^b^Tertile 1: n = 29; mean = 31.65 ± 7.77 min, 19.29 to 42.80; Tertile 2: *n* = 29; mean = 51.38 ± 5.17 min, 42.85 to 60.29; Tertile 3: *n* = 31; mean = 83.63 ± 25.49 min, 61.15 to 178.43

Table 4 shows the multivariable regression for group status and actigraphy results adjusted for age, depressive symptoms, years of education and BMI. *Total sleep time* showed a main effect for group (F(7, 82) = 372.2, *p* < 0.001, η2 = 0.969). MCI-DX group expressed shorter total sleep time than the NC group (*p* < 0.013). *Sleep fragmentation* showed a significant group effect (F (7, 82) = 88.0, *p* < 0.001, η2 = 0.883). Greater sleep fragmentation was observed for participants in the MCI-DX group than either NC group (*p* < 0.008) or MCI-CON group (*p* < 0.041). A main effect for *Sleep latency* (F (7, 82) = 11.5, *p* < 0.001, η2 = 0.496) was reflected by longer *sleep latency* in MCI-DX than MCI-CON groups (*p* < 0.008). Shorter *sleep latency* in MCI-CON group is evidence for sleep deprivation. *Sleep efficiency* (F (7, 82) = 1255.0, *p* < 0.001, η2 = 0.991) was poorer in MCI-DX than MCI-CON groups (*p* < 0.021). Main effect for group was found for *Wake time* (F (7, 82) = 104.5, *p* < 0.001, η2 = 0.899) and *mean activity* (F (7, 82) = 39.9, *p* < 0.001, η2 = 0.773). Higher wake time (*p* < 0.036) and mean activity (*p* < 0.006) were found in MCI-CON than NC groups.

**Table 4:** Nocturnal Actigraphy in MCI-DX, MCI-CON and NC Groups

Actigraphy Variable	MCI-DX	MCI-CON	NC	*p*
Wake Bout Time (WBT, min)	2.67	2.79^a^	2.32^a^	0.036^a^
Sleep Fragmentation (SF, freq)	45.75^a,b^	35.62^b^	32.79^a^	0.008^a^; 0.041^b^
Total Sleep Time (TST, min)	379.94^a^	431.89	454.30^a^	0.01^a^
Sleep Efficiency (SE, %)	76.74^a^	83.65^a^	82.01	0.021^a^
Sleep Latency (SL, min)	44.08^a^	13.84^a^	27.85	0.008^a^
Mean Activity Score (MA, freq)	13.63	18.67^a^	12.76^a^	0.006^a^

*Note*. Multivariable Regression of MCI status and Actigraphy (mean = 6.7 nights) measures adjusted with covariates age, current depressive symptoms, years of education, and BMI were significant for these parameters WBT, SF, TST, SE, SL, M.A. (*p* < .000); post-hoc group differences are shown (*p* < .001-.05) with superscript letters (a,b) reflect pairwise differences. No group findings emerged for time in bed, actual sleep percent, actual wake percent, sleep bouts, wake bouts, sleep bout time, immobile percent time, number of minutes moving, moving percent time, number of immobile phases, mean length immobility, mean one minute immobility, total activity score, wake after sleep onset (WASO), S.D. of activity counts, or largest activity count

Sleep questionnaires were examined for group differences using multivariable regression adjusted for age, education, depressive symptoms and BMI. The Pittsburg Sleep Questionnaire Inventory (PSQI) yields a composite score and components: sleep quality, sleep latency, sleep duration, sleep efficiency, sleep disturbances, use of sleep medications and daytime dysfunction. The model did not identify any significant group differences. However, the depressive symptoms covariate predicted PSQI composite (*p* < 0.012, η2 = 0.07), sleep disturbance (*p* < 0.001 η2 = 0.114) and daytime dysfunction (*p* < 0.001, η2 = 0.190), and BMI predicted daytime dysfunction (*p* < 0.026, η2 = 0.056). Epworth Sleep Scale (ESS) did not find any MCI group or covariate differences (p’s > 0.06-0.58), and Stanford Sleepiness Scale *(*SSS), completed on seven consecutive days, had poor participant compliance in 9 of 17 MCI-DX group participants. Notably, greater depressive symptoms was associated with higher SSS rating (*p* < 0.002, η2 = 0.12) [55].

### SM-RR coupling

During waking, movements induce increased respiratory rate that supports oxygen demands [56], but, to our knowledge, has not been studied systematically during sleep. In our work with the mattress device and paradigm, SM vigor (e.g., amplitude, area under the curve, AUC) is significantly reduced during the reduced arousability caused by sleep loss associated with neurological disease [37–40]. For the SM-RR coupling analysis using artificial intelligence (AI), we selected all cases from the MCI-DX group and participants with the lowest scores on the memory tests from the MCI-CON group (*n* = 3) to comprise the MCI/AI group (*n* = 20) and a randomly selected subset from the NC group termed NC/AI (*n* = 20). Using time series correlational analyses, the time latency estimates derived from 10 min segmentation windows across the 2 nights were examined. As described, FFT and digital filters identified two data streams at cutoff frequencies of 0.15 Hz (SM) and 0.37 Hz (RR). Time latency calculations were determined based on identifying events with respiratory frequency change based on a 1 S.D. threshold. Typically, our observations have found that SM precedes RR change (SM → RR). However, for some coupling events, RR preceded SM (RR → SM) and were calculated in the time window. Hence, time latencies may be positive or negative based on whether SM occurred before ( +) or after RR change (-).

**Fig. 3:**
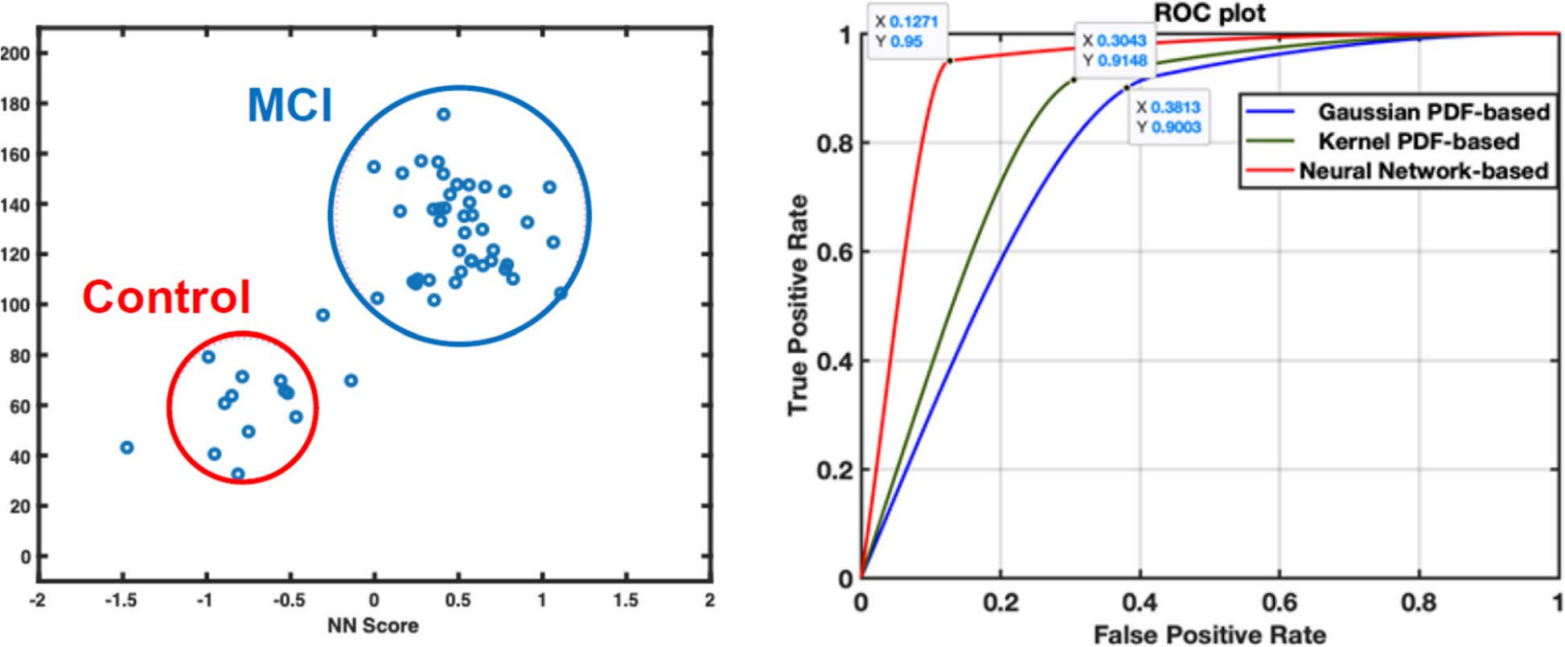
**A** Time Lag vs Neural Network Score for MCI and NC groups with the threshold cutoff value of 70 ms. **B** ROC plot comparing Gaussian, Kernel, and Neural Network based methods

The time latency algorithm was tested in the AI analysis as a classifier of MCI risk. Using the cross validation LOOC statistic, the training data set was developed and tested with several validation methods (e.g., Neural Networks, Gaussian and Kernel methods) described in Khosroazad et al. [41]. Figure 3a shows the distribution of time latency using the Neural Network approach where zero is the threshold, 70 ms. Note that the clusters of data points separate MCI from NC cases. We note that the time latency values associated with increased MCI risk were negative, i.e. showed the atypical pattern in which RR change leads SM events. For the ROC diagnostics shown in Fig. 3b., the 70 ms time latency threshold optimized detection of true MCI positive cases (sensitivity) vs. false positives (specificity). The ROC achieved an AUC of 88% sensitivity and 87% specificity.

## Discussion

Much recent work demonstrates that sleep and circadian rhythm disturbances in aging-related cognitive decline are clinically significant [57, 58], and are driven by complex neurodegenerative changes, stress-related cardiorespiratory and autonomic dysregulation, brain inflammation and incomplete β amyloid and tau CSF clearance [26, 27, 35, 36]. Of those who report aMCI symptomatology (i.e., memory loss, cognitive fog, etc.) to their healthcare provider, those who progress to AD are conservatively close to 10% per year [58, 59].

Progressing from primary care to early diagnosis in AD has advanced significantly through referral for screening and analysis of Aβ and tau biomarkers in brain, CSF and blood samples increasing the probability of early diagnosis and inclusion in therapeutic trials [10–14]. In the current study, the MCI-DX group met both biomarker and cognitive phenotype criteria for early AD and showed the greatest impairment in mattress and actigraphic sleep measures. In cognitive screening retest, the MCI-DX group met cognitive impairment criteria on the Montreal Cognitive Assessment (MoCA) with selective impairment in the memory domain (e.g. immediate and delayed recall in verbal and visuospatial memory), but not in intelligence or cognitive reserve measures compared to the community participants (MCI-CON and NC). The MCI-CON group scored at an intermediate level between the MCI-DX and NC groups in the memory domain and in sleep quality. These results are consistent with recent findings from the UK biobank on pre-diagnostic AD which had been determined by biomarkers compared to cognitive performance 4–9 years previously. Deficits in fluid intelligence, pairs matching and prospective memory were notably impaired in pre-diagnostic AD in comparison to frontotemporal dementia, progressive supranuclear palsy, dementia with Lewy bodies, Parkinson’s or multiple system atrophy [12]. Nonetheless, the fundamental differences between the two MCI groups in the current study may reflect a selection bias of who seeks medical care, and represents a limitation of the study.

To confirm the association cognitive performance and sleep disorder, we used seven days of actigraphy in the home bed. Cohort analyses identified that sleep fragmentation and wake after sleep onset *(*WASO) were associated with poorer scores on memory tasks, but sleep disorder was not associated with intelligence or cognitive reserve measures, confirming that memory-based cognitive decline was uniquely associated with poor sleep measures. To further examine this link in group analyses, MCI-DX revealed the clearest evidence of sleep disturbance, e.g., increased sleep fragmentation, increased sleep duration, and a trend for longer sleep latency, when compared to the NC group. MCI-CON group expressed less severe sleep disturbance: increased wake bout duration, mean activity and a trend for shorter sleep latency, a marker of cumulative sleep loss*,* compared to NC group. These findings confirm the seminal work of Ancoli-Israel et al. regarding actigraphic sleep fragmentation and WASO in nursing home patients with dementia [60]. In community dwelling older adults, Spira et al. found longer sleep duration, a marker of poor sleep quality, predicted poorer scores on memory, semantic fluency, and subjective cognitive problems [61]. Recent work has found that age-related cognitive loss correlated with reduced REM sleep, a critical sleep stage in processing of memory-related neuroplasticity [62].

The second hypothesis of the study inquires whether home sleep recordings and application of the biometric algorithm may offer a possible Alzheimer’s risk signature, and provide clinical indication for biomarker testing referral in the diagnosis of aMCI/early AD. Home sleep recordings using the novel movement-respiratory biometric proposed in this study may facilitate diagnosis of aMCI, and improve delivery of interventions for sleep and cognitive symptoms, although identification of an MCI sleep phenotype at the individual level has not yet been achieved with this pilot study. The AI classification algorithm and home mattress system may provide a useful biometric for assessing individual risk for MCI risk in aging adults. We have previously shown in other clinical samples that the movement arousal measured by the SM biometric can track suppressed arousability in sleep deprivation in chronic sleep disorder, a common problem in neurological conditions [37–43, 55]. For the AI classification test, patients with diagnosed aMCI and a community sample with normal cognition were tested for the fast (70 ms) time latency coupling linkage observed between SM bursts and respiratory upregulation. Diagnosed aMCI/AI group had consistently higher Neural Network scores (i.e., below the 70 ms cutoff threshold), and showed coupling reversal (i.e., RR → SM) reminiscent of movement arousals that follow apneic pauses in obstructive sleep apnea (OSA). OSA is common in aging and associated with cognitive deficits [63] and was not exclusionary, although rare: there were four cases in each AI group and all claimed compliance with CPAP treatment. ROC findings confirm the hypothesis that the SM → RR coupling reflects a highly predictive measure of novel autonomic features not captured by actigraphy or polysomnography. Our recent work regarding the attenuation of SM vigor with CNS impairment promoting sleep loss, and the robustness of respiratory coupling [41–43], suggests that SM periodicity may provide a neuroprotective mechanism against tissue hypoxemia during sleep that may be impaired in early AD. However, a significant limitation of the study is that we do not have longitudinal data on our study cohort, particularly, with relationship to changes in OSA and these measures over time. Future work will pursue the time latency hypothesis longitudinally and in larger samples to further explore the relationship of the SM → RR coupling to sleep apnea. In addition, we will study time latency changes longitudinally in the consensus MCI group to assess potential disease-related emergent properties over time.

In the present study, we observed a relationship between micro-sleep movements and respiratory variability showing respiratory upregulation is coupled to micro-movement arousal events. Periodic respiratory upregulation during sleep could support neuro-perfusion during consolidated sleep when autonomic control of respiratory drive is low [64]. We have shown that chronic sleep loss will reduce SM vigor which, in the present study, was found to inefficiently drive respiratory upregulation. We propose that during early AD, and perhaps, in other neurological diseases, this respiratory mechanism becomes impaired, and may lead to suboptimal oxygenation of neural tissue during sleep, a potential pathway to cognitive impairment in aging.

## Data Availability

The datasets generated and/or analyzed during the current study are not publicly available due to our intention to present these data to the Food and Drug Administration for consideration of our method of MCI detection. Additionally, these data form the basis of a new patent in preparation. But, the data can be made available from the corresponding author on reasonable request.
